# Should Reward Deficiency Syndrome (RDS) Be Considered an Umbrella Disorder for Mental Illness and Associated Genetic and Epigenetic Induced Dysregulation of Brain Reward Circuitry?

**DOI:** 10.3390/jpm12101719

**Published:** 2022-10-14

**Authors:** Kenneth Blum, Catherine A. Dennen, Igor Elman, Abdalla Bowirrat, Panayotis K. Thanos, Rajendra D. Badgaiyan, B. William Downs, Debasis Bagchi, David Baron, Eric R. Braverman, Ashim Gupta, Richard Green, Thomas McLaughlin, Debmalya Barh, Mark S. Gold

**Affiliations:** 1 The Kenneth Blum Behavioral & Neurogenetic Institute, LLC., Austin, TX 78701, USA; 2 Center for Behavioral Health & Sports, Exercise, Psychiatry, Western University Health Sciences, Pomona, CA 91766, USA; 3 Institute of Psychology, ELTE Eötvös Loránd University, Kazinczy u. 23–27, 1075 Budapest, Hungary; 4 Department of Molecular Biology and Adelson School of Medicine, Ariel University, Ariel 40700, Israel; 5 Department of Family Medicine, Jefferson Health Northeast, Philadelphia, PA 19140, USA; 6 Medicine, Boston Children’s Hospital, Boston, MA 02115, USA; 7 Cambridge Health Alliance, Harvard Medical School, Cambridge, MA 02139, USA; 8 Behavioral Neuropharmacology and Neuroimaging Laboratory, Department of Pharmacology and Toxicology, Jacobs School of Medicine and Biomedical Sciences, Clinical Research Institute on Addictions, University at Buffalo, Buffalo, NY 14203, USA; 9 Department of Psychology, University at Buffalo, Buffalo, NY 14260, USA; 10 Department of Psychiatry, South Texas Veteran Health Care System, Audie L. Murphy Memorial VA Hospital, Long School of Medicine, University of Texas Medical Center, San Antonio, TX 78229, USA; 11 Division of Precision Nutrition, Victory Nutrition International, LLC., Lederoch, PA 19438, USA; 12 Department of Pharmaceutical Science, College of Pharmacy & Health Sciences, Texas Southern University, Houston, TX 77004, USA; 13 Future Biologics, Lawrenceville, GA 30043, USA; 14 Centre for Genomics and Applied Gene Technology, Institute of Integrative Omics and Applied Biotechnology, Nonakuri, Purba Medinipur 721172, India; 15 Department of Psychiatry, Washington University School of Medicine, St. Louis, MO 63110, USA

**Keywords:** Reward Deficiency Syndrome (RDS), hypodopaminergia, Genetic Addiction Risk Severity (GARS) test, pro-dopamine regulation (KB220Z), dopamine homeostasis, epigenetics, brain reward cascade, addiction

## Abstract

Reward Deficiency Syndrome (RDS) is defined as a breakdown of reward neurotransmission that results in a wide range of addictive, compulsive, and impulsive behaviors. RDS is caused by a combination of environmental (epigenetic) influences and DNA-based (genetic) neurotransmission deficits that interfere with the normal satisfaction of human physiological drives (i.e., food, water, and sex). An essential feature of RDS is the lack of integration between perception, cognition, and emotions that occurs because of (1) significant dopaminergic surges in motivation, reward, and learning centers causing neuroplasticity in the striato-thalamic-frontal cortical loop; (2) hypo-functionality of the excitatory glutamatergic afferents from the amygdala–hippocampus complex. A large volume of literature regarding the known neurogenetic and psychological underpinnings of RDS has revealed a significant risk of dopaminergic gene polymorphic allele overlap between cohorts of depression and subsets of schizophrenia. The suggestion is that instead of alcohol, opioids, gambling disorders, etc. being endophenotypes, the true phenotype is RDS. Additionally, reward deficiency can result from depleted or hereditary hypodopaminergia, which can manifest as a variety of personality traits and mental/medical disorders that have been linked to genetic studies with dopamine-depleting alleles. The carrying of known DNA antecedents, including epigenetic insults, results in a life-long vulnerability to RDS conditions and addictive behaviors. Epigenetic repair of hypodopaminergia, the causative basis of addictive behaviors, may involve precision DNA-guided therapy achieved by combining the Genetic Addiction Risk Severity (GARS) test with a researched neutraceutical having a number of variant names, including KB220Z. This nutraceutical formulation with pro-dopamine regulatory capabilities has been studied and published in peer-reviewed journals, mostly from our laboratory. Finally, it is our opinion that RDS should be given an ICD code and deserves to be included in the DSM-VI because while the DSM features symptomology, it is equally important to feature etiological roots as portrayed in the RDS model.

## 1. Introduction

Reward Deficiency Syndrome (RDS) involves many mental health disorders rather than being treated as a unique and independent mental illness. These disorders include a wide range of addictive, compulsive, and impulsive behaviors. RDS, also characterized as an octopus of behavioral dysfunction, refers to abnormal behavior caused by a failure of the reward neurotransmission cascade as a result of genetic and environmental (epigenetic) effects. RDS interferes with the usual satisfaction of human physiological drives, such as the experience of pleasure associated with food and water consumption and sexual reproduction [1]. Epigenetic repair of hypodopaminergia, the causative basis of addicitive behaviors, may involve precision DNA-guided therapy achieved by combining the Genetic Addiction Risk Severity (GARS) test with KB220, a nutraceutical formulation with pro-dopamine regulatory capabilities established in at least 50 clinical trials [2,3,4,5]. In fact, in our latest annotated bibliography that has been peer-reviewed, there are at least 50 studies involving positive clinical outcomes based on various types of trials [5].

Although independent additional studies are merited to provide further validation, a large volume of literature regarding the known neurogenetic and psychological underpinnings of RDS has revealed a significant risk of dopaminergic gene polymorphic allele overlap between cohorts of depression and subsets of schizophrenia [6]. The suggestion is that instead of alcohol, opioids, gambling disorders, or overeating being endophenotypes, the true phenotype is RDS [7,8]. Furthermore, RDS may be fundamental to species evolution and survival, with an array of many neurotransmitter-polymorphic loci influencing dopaminergic net functionality [9,10]. Unfortunately, most of the evidence in terms of genetic aspects has come from our laboratory, but its acceptance is slowly being embraced.

Although not yet in the Diagnostic and Statistical Manual for Mental Disorders (DSM), RDS refers to the breakdown of the reward neurotransmission and destructive behaviors initiated by the combination of environmental (epigenetic) influences and DNA-based neurotransmission deficits that interfere with the usual achievement of the satisfaction of human physiological drives such as food, water, and sex [11]. The foundation of the RDS concept was initially established by Blum et al., who discovered that the A1 (minor) allele of the D2 dopamine receptor (DRD2) gene is linked to severe alcoholism, smoking, obesity, and other substance and non-substance disordered behaviors [12,13]. The purpose of this article is to explore the RDS behavioral model and the potential for treatment it may provide. RDS is now a featured psychological disorder and the subject of global research with 229 listings in Pubmed (10-3-22), 47% of which are independent of Blum’s laboratory, and 1466 listings when using the term “reward deficiency”, with only 134 from Blum’s laboratory. Specifically, 91.4% of these articles are independent of Blum’s group.

The disease concept of alcoholism was first proposed by E.M. Jellinek in 1946 [14]. However, this concept was initially met with suspicion and was not generally accepted due to a lack of scientific support [14]. However, during this time, the scientific and medical community agreed, in part, that deficits or imbalances in brain chemistry, perhaps genetic in origin, contributed to the development of alcoholism and other addictions, although this ideology was still controversial [15]. According to Pickard, “Conceptual clarification and preliminary empirical assessment of the Brain Disease Model of Addiction (BDMA) recommends agnosticism about its validity and an openness to heterogeneity; in some cases, addiction may be a brain disease, in others not. Either way, addiction stigma can be combatted by fighting moralism about drugs and moralistic drug policies directly, as opposed to resting hopes on the brain disease label”. To this retort, we simply respond that, while some people do not carry any polymorphisms related to neurotransmitter dysfunction, especially those associated with causing imbalances of dopamine in the meso-limbic center of the brain, the carrying of known DNA antecedents, including epigenetic insults, results in a life-long vulnerability to overall RDS conditions and especially addictive behaviors. Undoubtably, this is a brain disorder, and agnosticism would negate the thousands of highly sophisticated studies published in high-quality journals. There is definitely heterogeneity, as discussed above.

In the early 1960s, the interrelatedness of reward circuitry and the prefrontal cortices of the brain was not well understood. The core neurotransmitters were unknown. Serotonin, GABA, dopamine, acetylcholine, were not well characterized, and endorphins were not a part of the scientific acumen. Following Blum’s initial conceptualization of RDS in 1995, Blum et al. used the Bayesian Theorem [16] to find that carriers of the DRD2 A1 allele had a predictive value of 74.4% for future RDS behaviors such as substance use disorder (SUD), obesity, and shopping addiction. The number of genes associated with reward has significantly increased over time, especially from large gene-wide association studies (GWAS) involving millions of subjects [17,18]. In fact, in terms of major depression, transcriptome-wide association research analyses of major depression indicated significant correlations with the expression of DRD2 in the nucleus accumbens (NAc) and NEGR1 in the hypothalamus, among others. However, our laboratory has adopted an alternative approach involving finite pathways as candidates instead [19].

The proper release of dopamine occurs as a result of the cascading interaction of second messengers and neurotransmitters inside the NAc and across numerous brain regions. These regions are involved in cognition (memory), recall, motivation, decision-making, pleasure, wellbeing, stress reduction, drug reinstatement, and cravings. The end result is to establish dopamine homeostasis (i.e., the usual happiness setpoint) in homo sapiens [20], which is reflected in the resting-state functional connectivity (rsFC) in neuroimaging studies [21].

## 2. RDS Criteria

In the neurophysiologic reward system, repeated frequent acute dopamine stimulation becomes chronic stimulation and leads to a dysfunctional hypodopaminergic state, rendering the reward system less responsive to natural reinforcers, a symptom of RDS [22]. The stimulation can be from euphorigenic substances, non-substances like gambling, or severe stressors like pain and anxiety. Chronic stimulation causes dopamine depletion (hypodopaminergia). Reward deficiency results from depleted or hereditary hypodopaminergia, potentially reflected in a host of personality traits and mental and medical disorders that have been associated in genetic studies with dopamine depleting alleles. These symptoms and disorders create the diagnostic criteria for RDS and include, but are not limited to, novelty-seeking, schizophrenia, obesity, chronic pain, posttraumatic stress disorder (PTSD), major depression, and attention deficit hyperactivity disorder, among others (Table 1) [23]. Based on only counting alleles, albeit showing high risk if an individual carries at least four total polymorphic alleles for drug misuse and seven for alcoholism as determined against the ASI-MV 111. In terms of total overall risk for RDS behaviors, we trichotomized the resultant measured possibilities to express Mild, Moderate, Severe, and Profound risk.

As alluded to above, reward deficiencies may also occur in the absence of dopaminergic stimulation by exogenous factors due to specific polymorphic alleles that alter the function of genes in the reward cascade. One example is the A1 allele of the D2 dopamine receptor gene that causes a reduced number of dopamine receptors in the mesolimbic NAc [13,24]. An essential feature of RDS is the lack of integration between perception, cognition, and emotions occurring as a result of (1) significant dopaminergic surges in motivation, reward, and learning centers causing neuroplasticity in the striato-thalamic-frontal cortical loop, with ensuing top-down dissociation from the subcortical activity; (2) hypo-functionality of the excitatory glutamatergic afferents from the amygdala-hippocampus complex failing to generate bottom-up restraint of the striato-thalamic-frontal cortical loop [25,26].

Co-occurrences, similarities in the phenomenological and behavioral appearance, and empirical studies of some shared psychological and molecular mechanisms of addictive behaviors indicate a more integrative approach to the concept of addictive behaviors. The aim of one notable study was to investigate the possible genetic overlaps between different types of substance use, behavioral addictions, and other compulsive behaviors. A genetic association analysis was carried out as a part of the Psychological and Genetic Factors of Addictions (PGA) study [27] to assess several types of addictions in a sample of 3003 adolescent participants. The genetic association analyses focused on 32 SNPs, addictive substances (alcohol, tobacco, marijuana, and other illicit drugs), and potentially addictive behaviors (internet use, gaming, social media use, gambling, exercise, trichotillomania, and eating disorders). The association analysis revealed 29 nominally significant associations, of which nine survived the FDRbl correction for multiple testing. Four out of these nine significant associations were observed between a FOXN3 SNP and various addictions: rs759364 showed an association with the frequency of alcohol consumption and the mean scores of the internet use, gaming, and exercise addiction questionnaires. Significant associations have been found between GDNF rs1549250, rs2973033, CNR1 rs806380, and DRD2/ANKK1 rs1800497 variants and the “Lifetime Other drugs” variable [28].

In recent years, more and more scientific research emphasizes the overlaps between the symptomatology of different types of addictions. Theoretical models of addictive disorders handling “addictions” as a common disorder instead of distinct disorders have already been proposed in the 80s [29]. More recent research also encourages considering addiction not as a collection of different disorders [30], but as a symbolical umbrella under which all addiction types can be classified. Furthermore, the Obsessive-Compulsive Spectrum Disorder (OCSD) model by Hollander suggests a shared obsessive-compulsive spectrum in the background of psychiatric diseases of different diagnostic categories [31]. In addition, Blum proposed the concept of RDS in 1996, claiming that the development of impulsive and addictive behaviors shares some common psychological and molecular pathways [32]. Focusing on the phenomenological aspects, the Component Model of Addictions by Griffiths [33] argues that all addictions share six basic characteristics. Empirical studies underlie the concept of shared psychological and molecular mechanisms proposed in these models. For example, tolerance is one of the key criteria for many types of addiction as it takes a higher dose of the substance or behavior to achieve the same effect as earlier. Even a gambler may experience physical symptoms that are similar to either stimulant, opioid, or poly-substance withdrawal [34,35]. Furthermore, withdrawal symptoms, craving, impaired social, occupational, or recreational ability, and unsuccessful quitting attempts are key elements in many addictions, but the maintenance of these RDS behaviors may be a function of epigenetic insults [36,37].

The classification and the diagnostic criteria of addictive behaviors in the fifth edition of the DSM (DSM-5; American Psychiatric Association, 2013) and the eleventh revision of the International Classification of Diseases (ICD-11; World Health Organization, 2018) also reflect the phenomenological similarities of these behaviors. The revised DSM-5 contains the updated classification “Substance-related and Addictive Disorders”, replacing the “Substance-related Disorders” category from the DSM-IV-TR. In addition, gambling disorder is also included in the DSM-5 under the Non-Substance Related Disorders category of Substance-Related and Addictive Disorders. The new “Substance-related and Addictive Disorders” terminology in the DSM-5 is much more permissive in regard to behavioral addictions. The same trend can be seen in ICD-11, in which gambling and gaming disorders were both included under the classification of psychiatric disorders [38].

## 3. RDS Is a Behavioral Octopus

Results of family, twin, and adoption studies estimate the heritability (i.e., the overall genetic contribution) of addictions to be 50–70%, and more recently, with cannabis use disorder [39]. The brain’s reward system has a great impact on behavioral control and plays an important role in the pathophysiology of addictive behaviors. Dopaminergic and serotonergic neurotransmitter systems involved in these reward pathways are at the center of attention in candidate gene studies of substance abuse, non-substance addictions, and various risk behavior-related traits such as novelty seeking, impulsivity, or aggressive behavior [40,41]. Functional neuroimaging studies have shown that cocaine, money, and beauty similarly energize the reward circuitry of the brain [42], implying that similar neurobiological pathways are stimulated in the brain regardless of the object of the addiction [43]. The release of dopamine in the NAc during rewarding experiences provides reinforcement to motivate such activities. Serotonin modulates the reward pathway and is also involved in emotion and behavior regulation [44].

**Figure 1:**
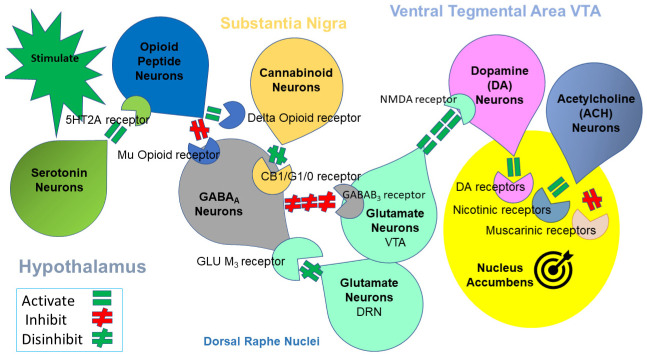
Mesolimbic Brain Reward Cascade. This cartoon illustrates the interaction of known major neurotransmitter pathways involved in the Brain reward Cascade (BRC). In the hypothalamus, environmental stimulation results in the release of serotonin, which in turn, via, for example, 5 HT-2 a receptors activate (green equal sign) the subsequent release of opioid peptides from opioid peptide neurons. Then, in the Substantia Nigra, the opioid peptides move to possibly two different opioid receptors with different effects. One that inhibits (red hash sign) through the mu-opioid receptor (possibly via enkephalin) to GABA_A_ neurons. Another stimulates (green equal sign) cannabinoid neurons (the Anandamide and 2-*arachidonoylglycerol,* for example) through beta-endorphin-linked delta receptors, which inhibit GABA_A_ neurons. Furthermore, when activated, cannabinoids, primarily 2-*arachidonoylglycerol*, can indirectly disinhibit (green hash sign) GABA_A_ neurons through activation of G1/0 coupled to CB1 receptors. In the Dorsal Raphe Nuclei (DRN), glutamate neurons can then indirectly disinhibit GABA_A_ neurons in the Substantia Nigra through activation of GLU M_3_ receptors (green hash sign). GABA_A_ neurons, when disinhibited, will in turn, powerfully (red hash signs) inhibit VTA glutaminergic drive via GABAB 3 receptors. At the Nucleus Accumbens, Acetylcholine (ACH) neurons may stimulate both muscarinic (red hash) and Nicotinic (green hash) receptors. Finally, Glutamate neurons in the VTA will project to dopamine neurons through NMDA receptors (green equal sign) to preferentially release dopamine at the Nucleus Accumbens (NAc), shown as a bullseye that indicates a euphoria, or “wanting” response. The result is dopamine release; low release is (endorphin deficiency), where unhappiness is felt. General (healthy) happiness depends on the dopamine homeostatic tonic set point (with permission) [22]. Notably, various hypotheses explained the findings that led to the modern known correlates of neurotransmitter interactions within this brain reward circuitry. Hypothalamus = serotonin and opioid peptides; Substantia Nigra = Cannabinoids, and GABA; Dorsal Raphe Nuclei = Glutamine and GABA; Ventral Tegmental Area(VTA) = Glutamine and acetylcholine, and Nucleus Accumbens = Dopamine.

Genetic factors may also influence how and when individuals develop addictive behaviors by affecting the sensitivity to drug effects at first use. However, it is further modified by epigenetic mechanisms, which could be prevalent for at least F2 generations [45]. In our opinion, although it may not be universally accepted as of yet, one of the broadly accepted hypotheses in the development of addiction is the RDS, postulating that decreased dopamine receptor density or sensitivity in the brain causes a weaker reward-sensation and, to compensate for it, individuals develop various types of behaviors to increase their dopamine levels [46]. We are cognizant that dopamine is not the singular cause of any addictive behavior and that it is indeed the interrelatedness of at least seven major neurotransmitter systems (see Figure 1) and possibly hundreds of second messengers. The syndrome consists of a variety of compulsive, addictive, and impulsive behaviors (i.e., drug addiction, compulsive eating, smoking, gambling, sex addiction, internet gaming). To be clear, it is indeed the interrelatedness of at least eight major neurotransmitter pathways working in concert to release dopamine at the NAc (Figure 1).

Mental health disorders are health conditions that cause changes in thinking, emotions, or behavior. Mental health disorders and mental illnesses are distinguished by the etiology of their conditions. Furthermore, mental illnesses are diagnosed by a medical professional and characterized as bodily diseases that significantly impair emotional, cognitive, or social abilities. Mental illnesses occur with varying severity and include mood disorders (i.e., anxiety, depression, and bipolar disorder), psychiatric conditions (i.e., personality disorders), and psychotic disorders (i.e., schizophrenia and eating disorders).

**Figure 2:**
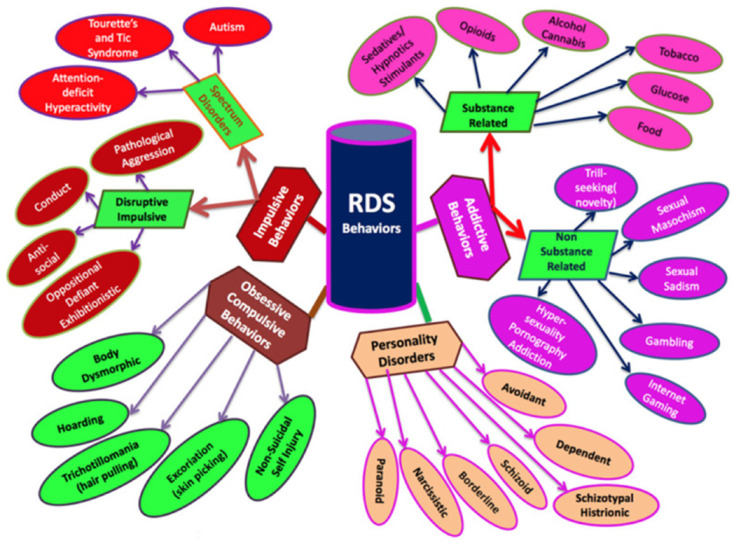
Reward Deficiency Syndrome schematic (with permission from Blum et al. [46]).

Currently, the DSM is the definitive resource for all mental disorders. The DSM-5 includes diagnostic criteria for SUD that distinguish between SUD and substance-induced disorders. SUD criteria are based on the harmful consequences of repeated use, but substance-induced disorders include intoxication, tolerance, compulsive use, and/or withdrawal. Concurrently, genetic covariance between substance and non-substance disordered individuals can be the result of individuals carrying reward gene allele variations (polymorphisms). Thousands of studies, cases, reviews, and meta-analyses show significant dopaminergic gene polymorphism overlaps between RDS and many psychiatric illnesses, although additional research is merited (Figure 2). This genetic covariance amongst individuals due to genetic polymorphisms can be utilized to predict phenotypic responses to environmental circumstances.

While actual data on the effect of KB220 variants on either epigenetic induced histone acetylation or methylation has not as yet been accomplished, indirect inference can be gleaned from the following summarized published reports. It is widely acknowledged that in both food and drug-addicted individuals, there is dopamine resistance because of an association with the DRD2 gene A1 allele [47,48,49]. Evidence is emerging for utilizing a natural, non-addicting, safe, putative D2 agonist as a means to recover from reward deficiency syndrome (RDS) for patients addicted to psychoactive chemicals [49]. As mentioned earlier, our laboratory has conducted a number of clinical trials, everything from triple-blinded to double-blinded placebo-controlled, randomized controlled trials, neuroimaging studies, case series, and other notable trials (see [5]). The following retort briefly summarizes a number of these studies, all peer reviewed and published to date. The KB220 variant had a number of variants with different trade names since its basic inception in the late 70 s, which unfortunately is confusing.

Quantitative electroencephalography (qEEG) as an imaging tool revealed the impact of Synaptamine Complex Variant KB220 containing DLPA as a putative activator of the mesolimbic system [48,49,50]. Other work utilizing the KB220 variant, administered via an intravenous injection, decreases or “normalizes” aberrant electrophysiological parameters of the reward circuitry site [51]. In that published pilot study [51], it has been found that the qEEG’s of a heroin abuser and an alcoholic demonstrate abnormalities (widespread alpha and theta activity, respectively) during protracted abstinence; however, their qEEG’s were significantly normalized by the administration of 1 intravenous dose of Synaptamine Complex Variant KB220Z. Specifically, both patients were genotyped for a variety of neurotransmitter reward genes to ascertain the extent of putative dopaminergic risk alleles they carry that may predispose them to heroin or alcohol dependence, respectively. The tested genes included the dopamine transporter (DAT1, locus symbol SLC6 A3), DRD2 TaqIA (rs1800497), dopamine D4 receptor exon 3 VNTR (DRD4), monoamine oxidase A upstream VNTR (MAOA-uVNTR), serotonin transporter linked polymorphic region (5 HTTLPR, locus symbol SLC6 A4), and COMT val158 met SNP (rs4680). Generally, the patients in this latter study showed a DNA antecedent of hypodopaminergia. These genetic results provide some rationale to support the concept of the induction of “dopamine homeostasis”. Moreover, this initial work is supported by a clinical trial on Synaptamine Complex Variant KB220 utilizing intravenous administration in more than 600 alcoholic patients, resulting in significant reductions in RDS behaviors [52]. In addition, Blum’s laboratory evaluated the natural dopaminergic agonist, KB220 intravenous (IV), and its oral variants, to enhance dopaminergic function in SUD. Our pilot experiment revealed a significant reduction in chronic symptoms, measured by the Chronic Abstinence Symptom Severity (CASS) Scale. The combined group (IV and oral) did significantly better than the oral-only group over the first week and a 30-day follow-up period. Following this, the combination was given to 129 subjects, and three factors were measured: emotion, somatic, and impaired cognition. Each had eigenvalues larger than one, and they were extracted for baseline CASS-Revised (CASS-R) variables. Paired sample t-tests for pre and post-treatment scales showed significant reductions (*p* = 0.00001) from pre-treatment to post-treatment. The values were t = 19.1 for emotion, t = 16.1 for somatic, and t = 14.9 for impaired cognition. A two-year follow-up of 23 subjects who experienced KB220 IV therapy, which includes at least five IV treatments over seven days and oral treatments for 30+ days, revealed that 21 (91%) of the subjects were sober at six months and 19 (82%) had no relapse. 19 (82%) subjects were sober at one year, and 18 (78%) had no relapse. 21 (91%) subjects were sober two years post-treatment, and 16 (70%) had no relapse [53].

There is evidence that modifications in synchronous neural activity between brain regions involved in reward and other cognitive functions may significantly contribute to substance-related disorders. Our previous work by Febo et al. [54] provided the first evidence demonstrating that the pro-dopaminergic nutraceutical KB220Z significantly increases (above placebo) the functional connectivity between reward and cognitive brain regions in rats. The following regions were affected: the nucleus accumbens, anterior cingulate gyrus, anterior thalamic nuclei, hippocampus, and prelimbic and infralimbic loci. In addition, significant functional connectivity, increased brain connectivity volume recruitment (potentially neuroplasticity), and dopaminergic functionality were found across the brain reward circuitry. Most importantly, increases in functional connectivity were specific to these regions and were not broadly dispersed across the brain.

Furthermore, our laboratory also showed the effect of KB220Z on reward circuitry for ten heroin addicts who were undergoing protracted abstinence (on average 16.9 months). In a placebo-controlled, randomized crossover study of KB220Z, five subjects participated in a triple-blinded experiment where the subject, the person evaluating the response to treatment, and the person administering the treatment were all blinded to the treatment that any individual subject was receiving. Additionally, nine subjects were genotyped utilizing the Genetic Addiction Risk Score (GARS) test. Previously, Blum’s group [55] preliminarily reported that KB220Z induced an increase in BOLD activation in caudate-accumbens-dopaminergic pathways relative to placebo, following a one-hour acute administration. Moreover, KB220Z also decreased resting-state activity in the putamen of abstinent heroin addicts. In the second phase of this pilot study, we noted that three brain regions were significantly activated from the resting state by KB220Z relative to placebo (*p* < 0.05) for all 10 abstinent heroin-dependent subjects. Enhanced functional connectivity was observed in a putative network, which includes the medial frontal gyrus, nucleus accumbens, posterior cingulate, occipital cortical areas, cerebellum, and dorsal anterior cingulate.

These results, along with other quantitative electroencephalography (qEEG) study results, suggest a putative [56] anti-craving/anti-relapse role of KB220Z in psychostimulant addiction by direct or indirect dopaminergic interaction. Recently, Willuhn et al. [57] reported that cocaine use, as well as non-substance-related addictive behaviors, increases as dopaminergic function is decreased. Chronic cocaine exposure has been associated with reductions in D2/D3 receptors and lower activation of cues in the occipital cortex and cerebellum, which was found in a PET study by Park et al. [58] Therefore, treatment strategies, such as dopamine agonist therapy that conserves dopamine function, could be an interesting approach to the prevention of relapse in psychoactive drug and behavioral addictions.

Importantly, there are a number of compounds that have been shown to inhibit the degradation of enkephalins [58]. Enkephalinase inhibitors (EIs) appear to be promising as therapeutic agents due to their analgesic properties, which are accomplished by increasing enkephalins. Endogenous EIs include peptides like opiorphin and spinorphin. Endogenous and synthetic inhibitors of enkephalin degrading enzymes have been studied in vivo utilizing standard animal models [59,60]. It is currently known that these enkephalinase inhibitors prevent the degradation of enkephalins, whereby these compounds produce naloxone reversible analgesia and potentiate the analgesia produced by enkephalins and acupuncture. D-phenylalanine has been shown to help many individuals suffering from chronic, intractable pain [61,62]. Blum’s group [47] proposed that enkephalinase inhibitors may be effective in a number of people with “endorphin deficiency diseases” such as depression, schizophrenia, convulsive disorders, and arthritis. Cheng et al. [62] revealed that the D-amino acids (DAA), diphenylalanine, and D-leucine produce naloxone reversible analgesia; electroacupuncture (EA) also produces analgesia, which is blocked by naloxone. Combining the two treatments produces an additive effect with greater analgesia than that produced by either treatment given alone. In addition, EIs may alleviate other conditions associated with decreased endorphin levels, such as alcohol/opiate withdrawal symptoms [63]. In the Blum et al. [63] review article, they point out that the consensus of the literature supports the concept that brain neurotransmitters and second messengers are involved in the net release of dopamine in the mesolimbic region, especially the nucleus accumbens (NAc). Furthermore, the release of neuronal dopamine is directly linked to motivation, anti-stress, incentive salience (wanting), and well-being. The role of dopamine in terms of alcohol withdrawal symptomology, cocaine-craving behavior, dopamine-condensation products (TIQs), and, more recently, the genetic aspects of drug-seeking and pro-dopamine regulation, provides compelling evidence of the relevant molecular neurological correlates of dopaminergic/endorphinergic mechanisms in reward circuitry due to genetic polymorphisms and epigenetic insults.

Certainly, in the face of the American opioid epidemic, the clinical consensus is to treat opioid use disorder (OUD) with life-long opioid substitution therapy. However, it has been suggested that a paradigm shift involving novel modalities, such as targeting the endorphinergic system linked to dopamine release at the NAc, leads to the induction of “dopamine homeostasis [64]”. Utilizing the known gene-environment interaction theorem P = G + E, Blum et al. [64] previously provided a clear rationale for the adoption of genetic risk testing coupled with endorphinergic/dopamine regulation to address dysfunction across the brain reward circuitry. The goal of altering resting-state functional connectivity may require a gentle “neurotransmitter fix” via enkephalinase inhibition (e.g., D-phenylalanine) to overcome self-induction of acute dopamine release via psychoactive substance misuse, resulting in chronic dopamine downregulation.

As subsets of reward deficiency, we are poised to provide novel, genetically guided therapy for endorphinergic, opioidergic, and dopaminergic deficiencies and related syndromes, utilizing “Precision Addiction Management”. In terms of the therapeutic benefits of enkephalinase inhibition, there have been a series of articles that have focused on the role of endorphins in alcoholism [29]. One of these articles revealed that alcohol consumption significantly decreases leuenkephalin synthesis in brain circuits [65]. In a related article, it was also found that alcohol intake in genetically bred ethanol-preferring or ethanol-averse mice was found to be an inverse function of the amount of brain methionine-enkephalin (METENK) present [66].

Simply, the lower the amount of brain methionine-enkephalin, the higher the intake of alcohol in C57/Bl mice (low METENK), and the lower the intake of alcohol in DBA mice (high METENK). Using these studies as a rationale, Blum et al. [67] performed the first pharmacogenetic engineering experiment employing D-Phenylalanine, the enkephalinase inhibitor, to convert ethanol-preferring C57/Bl mice to behave like ethanol-averse DBA mice. These authors were able to significantly attenuate both volitional and forced ethanol intake, respectively, by acute and chronic treatment with D-phenylalanine. D-phenylalanine raises brain enkephalin levels through its enkephalinase inhibitory activity. It was shown that 18 days of treatment with D-phenylalanine significantly attenuated excessive alcohol intake in C57/BL to the same or even lower levels than its counter-part, DBA mice. This suggests that alcohol intake can be regulated by the alteration of endogenous brain opioid peptides.

Blum’s laboratory also researched the effects of the amino acid and vitamin mixture SAAVE in inpatient, chemically dependent patients in order to assess the role of neurotransmitters in assisting with the recovery and adjustment to a detoxified, sober state. In a double-blind, placebo-controlled, randomized study of 62 alcoholics and polydrug abusers, SAAVE patients had a significantly reduced stress response as measured by the skin conductance level (SCL). In addition, SAAVE patients had significantly improved physical scores and BESS scores (behavioral, emotional, social, and spiritual). Following detoxification, a six-fold reduction in AMA rates was seen when comparing SAAVE vs. placebo groups. In this inpatient treatment experience, the BESS scores indicated that SAAVE aided in the rate of recovery and helped patients respond more effectively and swiftly to the program’s behavioral goals [68].

According to a study involving Driving-Under-the-Influence (DUI) offenders by Brown et al. [69], the central nervous system rewarding properties of ethanol, heroin, and cocaine might stimulate a common catecholaminergic reward system in the mesolimbic circuitry of the brain. DUI offenders with either alcohol or cocaine-related issues were investigated. The KB220 neuronutrients SAAVE and Tropamine significantly reduced relapse rates and enhanced recovery in these DUI outpatient offenders over a 10-week period. Follow-up on both the SAAVE and Tropamine groups after 10 months revealed a 73% and a 53% overall recovery rate, respectively.

Understanding that there is no FDA approved treatment for psychostimulant abuse, research was conducted on abstinent psychostimulant abusers [57]. Positive outcomes demonstrated by quantitative electroencephalographic (qEEG) imaging in a randomized, triple-blind, placebo-controlled, crossover study involving oral Synaptose Complex KB220Z showed an increase in alpha waves and low beta wave activity in the parietal brain region. Using t statistics, significant differences observed between placebo and Synaptose Complex KB220Z consistently occurred in the frontal regions after week 1 and then again after week 2 of analyses (*p* = 0.03). This is the first report to demonstrate involvement of the prefrontal cortex in the qEEG response to a natural putative D2 agonist (Synaptose Complex KB220Z), especially evident in dopamine D2 A1 allele subjects. Independently, we have further supported this finding with an additional study of 3 serious polydrug abusers undergoing protracted abstinence who carried the DRD2 A1 allele. Significant qEEG differences were found between those who received 1 dose of placebo compared with those who were administered Synaptose Complex KB220Z. In these addicts, Synaptose Complex KB220Z induced positive modulation of their brain’s dysregulated electrical activity. The results are indicative of a phase change from low amplitude or low power in the brain to a more regulated state by increasing an average of 6.169 mV^2^ across the prefrontal cortical region. In the first experiment, we found that while 50% of the subjects carried the DRD2 A1 allele, 100% carried ≥1 risk allele. Specifically, based on the proposed addiction risk score for these 14 subjects, 72% had moderate-to-severe addiction risk. Comparable findings were acquired by replicating the experiment in 3 additional currently abstinent polydrug abusers who were carriers for the DRD2 A1 allele.

## 4. Summary

While we believe that in the future, with more independent research by other scientists, RDS deserves to be included in the DSM-VI and should be given an ICD code, we are also cognizant that the brain is not carved out according to the DSM. In this regard, Hyman’s group discussed this issue [70]. Specifically, neuroscience research into psychiatric disorders typically relies on disease classifications that are established by the influential DSM. The DSM was designed solely as a diagnostic tool, and it treats different disorders as distinct entities. However, the boundaries between disorders are not always as clear as the DSM claims. The US National Institute of Mental Health (NIMH) created the Research Domain Criteria (RDoC) project to provide an alternative framework for research into psychiatric disorders. There are five “domains” in the RDoC, and each reflects a brain system in which functioning is impaired, to varying degrees, in various psychiatric conditions. In agreement with these concepts, it is our opinion that while the DSM features symptomology, it would be equally important to feature etiological roots as portrayed in the RDS model [71,72,73].

In terms of a balanced review, a recent PUBMED search using the term “negative reports on Reward Deficiency Syndrome (RDS)” as of 10-3-22 revealed only four listings. However, using the term “reward deficiency”, there are 32 listings. A review of these entries does not reveal any direct negativity towards the concept of RDS per se. Furthermore, we are cognizant that certainly in some cases, there is a surfeit rather than a deficit of dopaminergic activity, especially in adolescence. Specifically, Blum et al. [72] suggest that the risk of all addictive drug and non-drug behaviors, especially in the unmyelinated prefrontal cortex of adolescents, is important and complex. Many animal and human studies show the epigenetic impact on the developing brain in adolescents compared to adults. Some reveal an underlying hyperdopaminergia that seems to set our youth up for risky behaviors by inducing high quanta of pre-synaptic dopamine release at reward site neurons. In addition, altered reward gene expression in adolescents caused epigenetically by social defeat, like bullying, can continue into adulthood. In contrast, there is also evidence that epigenetic events can elicit adolescent hypodopaminergia. This complexity suggests that neuroscience cannot make a definitive claim that all adolescents carry a hyperdopaminergic trait [73].

## 5. Conclusions

The carrying of known DNA antecedents, including epigenetic insults, results in a life-long vulnerability to RDS conditions and addictive behaviors. Epigenetic repair of hypodopaminergia, the causative basis of addictive behaviors, may involve precision DNA-guided therapy achieved by combining the GARS test with a researched neutraceutical having a number of variant names, including KB220Z [74]. This nutraceutical formulation with pro-dopamine regulatory capabilities has been studied and published in peer-reviewed journals mostly from our laboratory, but with required additional research, may help solve the current addiction crisis. Finally, following additional animal and human investigations, it is our opinion that RDS should be given an ICD code and deserves to be included in the DSM-VI because while the DSM features symptomology, it is equally important to feature etiological roots as portrayed in the RDS model.

## Figures and Tables

**Table 1:** Reward Deficiency Syndrome Criteria.

GARS Score of 4	With 1–2 Criteria	Mild
GARS Score of 4–6	with 3 or more Criteria	Moderate
GARS Score of 7–9	with 1–2 Criteria	Moderate
GARS Score of 7–9	with 3 or more Criteria	Severe
GARS Score of 10 or more	with 1–2 Criteria	Severe
GARS Score of 10 or more	with 3 or more Criteria	Profound
**Criteria Set ONE—DSM-5 Disorders**		
**A present diagnosis, past diagnosis, or history of these behavioral disorders**		
Substance Use Process Disorders	**Disorders:** Alcohol Use Disorder, Opioid Use Disorder, Cannabis Use Disorder; Sedative, Hypnotic, Anxiolytic Use Disorder; Cocaine Use Disorder, Amphetamine Use Disorder, Hallucinogen Use Disorder, Nicotine Use Disorder, Inhalant Use Disorder, Other, Unknown Substance Use Disorder	
	*Specifiers: Mild, Moderate, Severe, Early Remission (6–12 months), Sustained Remission (12+ months), in a Controlled Environment, on Maintenance Therapy*	
Process Disorders	Gambling, Sex, Other Specified Process Disorders	
Depressive (and related) Disorders	Major Depression, Dysthymia, Disruptive Mood Dysregulation, SUD/Medication/Medical Condition Induced Depressive Disorder, Disruptive Premenstrual Dysphoric Disorder	
Anxiety Disorders	Generalized Anxiety Disorder, Social Anxiety, Panic Attack	
	Disorder, Separation Anxiety, Selective Mutism, Specific Phobia,	
	SUD/Medication/Medical Condition Induced Anxiety	
Trauma and Stress Disorders	Reactive Attachment, Disinhibited Social Engagement, Post-Traumatic Stress Disorder (PTSD), Acute Stress Disorders	
Disruptive, Impulse Control, and Conduct Disorders	Oppositional Defiant Disorder, Intermittent Explosive Disorder, Conduct Disorder, Pyromania, Kleptomania	
Personality Disorders	General Personality Disorder, Paranoid Personality Disorder, Schizoid/Schizotypal Personality Disorder, Anti-Social Personality Disorder, Borderline Personality Disorder, Histrionic Personality Disorder, Narcissistic, Personality Disorder, Avoidant Personality Disorder, Dependent Personality Disorder	
Obsessive Compulsive Disorders and Related Disorders	Trichotillomania, Excoriation Disorder, SUD/Medical/Medication Induced Obsessive Compulsive Disorder (OCD), other Medical Condition, Induced Personality Disorder	
Schizophrenic Disorders	**Schizophrenic Disorders:** Schizophrenia, Schizoaffective Disorder, Schizophreniform Disorder, Delusional Disorder, Brief Psychotic Disorder, MH/Medical Catalonia, SUD/Medication/Medical Condition Induced Psychotic Disorder	
Dissociative Disorders	Dissociative Identity Disorder, Dissociative Amnesia, Depersonalization/Derealization Disorder	
Other Not Otherwise Specified (NOS) Disorders	Gender Dysphoric Disorder	
	Paraphilic Disorders	
Spectrum Disorders	Attention Deficient Disorder, Attention Deficient/Hyperactivity Disorder, Tourette’s Syndrome Autism	
**Criteria Set TWO**		
**Reported history of these symptoms:**		
Novelty seeking	This trait is associated with exploratory activity in response to novel stimulation, impulsive decision making, extravagance in approach to reward cues, quick loss of temper, and avoidance of frustration.	
Impulsivity	The construct of impulsivity includes at least two independent components: first, acting without an appropriate amount of deliberation, which may or may not be functional; and second, choosing short-term gains over long-term ones.	
Difficulty feeling reward (Anhedonia)	Either a reduced ability to experience pleasure or a diminished interest in engaging in pleasurable activities.	
Motivational Anhedonia	Decrease in motivation to participate in pleasurable activities	
Rumination, Obsessive, and Intrusive Negative Thoughts	Possible causes and consequences, as opposed to its solutions	

## Data Availability

Not applicable.
